# Germline Variation in Cancer-Susceptibility Genes in a Healthy, Ancestrally Diverse Cohort: Implications for Individual Genome Sequencing

**DOI:** 10.1371/journal.pone.0094554

**Published:** 2014-04-11

**Authors:** Dale L. Bodian, Justine N. McCutcheon, Prachi Kothiyal, Kathi C. Huddleston, Ramaswamy K. Iyer, Joseph G. Vockley, John E. Niederhuber

**Affiliations:** Inova Translational Medicine Institute, Inova Health System, Falls Church, Virginia, United States of America; IFOM, Fondazione Istituto FIRC di Oncologia Molecolare, Italy

## Abstract

Technological advances coupled with decreasing costs are bringing whole genome and whole exome sequencing closer to routine clinical use. One of the hurdles to clinical implementation is the high number of variants of unknown significance. For cancer-susceptibility genes, the difficulty in interpreting the clinical relevance of the genomic variants is compounded by the fact that most of what is known about these variants comes from the study of highly selected populations, such as cancer patients or individuals with a family history of cancer. The genetic variation in known cancer-susceptibility genes in the general population has not been well characterized to date. To address this gap, we profiled the nonsynonymous genomic variation in 158 genes causally implicated in carcinogenesis using high-quality whole genome sequences from an ancestrally diverse cohort of 681 healthy individuals. We found that all individuals carry multiple variants that may impact cancer susceptibility, with an average of 68 variants per individual. Of the 2,688 allelic variants identified within the cohort, most are very rare, with 75% found in only 1 or 2 individuals in our population. Allele frequencies vary between ancestral groups, and there are 21 variants for which the minor allele in one population is the major allele in another. Detailed analysis of a selected subset of 5 clinically important cancer genes, *BRCA1*, *BRCA2*, *KRAS*, *TP53*, and *PTEN*, highlights differences between germline variants and reported somatic mutations. The dataset can serve a resource of genetic variation in cancer-susceptibility genes in 6 ancestry groups, an important foundation for the interpretation of cancer risk from personal genome sequences.

## Introduction

Advances in sequencing technologies and decreasing costs are making whole genome sequencing (WGS) and whole exome sequencing (WES) increasingly accessible and may enable the transition from research applications and consumer genomics to routine clinical care. However, wide acceptance in the clinic has been hampered primarily by limitations in our current knowledge of the clinical relevance of the detected sequence variations.

In oncology, WGS/WES is currently used primarily to identify somatic mutations in tumors. Germline variations impacting cancer predisposition or disease progression are typically identified by targeted resequencing of genes of interest such as *BRCA1* and *BRCA2*. As WGS/WES becomes more widely adopted, analysis of germline variation will move from single-gene approaches to analyses based on multiple cancer-associated genes, and the tested population will expand from at-risk individuals to the general population.

The interpretation of these data requires an understanding of the variation in cancer risk-associated genes in healthy individuals, which is largely uncharacterized. Most knowledge of germline variation in cancer-susceptibility genes has come from individuals who have a medical reason to be sequenced [1], and so is not representative of the general population. Other information has come from cell lines and animal models rather than primary patient cells [2]. Individuals studied are primarily of European ancestry [1], [3], but both genome sequences and cancer risk vary between ancestry groups [4]. Furthermore, research studies have focused on high-penetrance susceptibility alleles, but cancer is generally the result of the combined effects of low- to moderate-penetrance risk alleles and environmental factors [5].

The goal of this study is to characterize the variation in cancer-susceptibility genes in a general population. To accomplish this goal, we profiled the nonsynonymous variation in 158 cancer genes using data from high-quality whole genome sequences from an ancestrally diverse cohort of 681 individuals. We also characterized in detail the variants in five genes of particular clinical interest, *BRCA1*, *BRCA2*, *KRAS*, *TP53*, and *PTEN*. The results can serve as a reference for variation in the 158 cancer-susceptibility genes in the general population and have important implications for the interpretation of clinical WGS/WES.

## Methods

### Ethics statement

Individuals were recruited at Inova Fairfax Hospital during 2011-2012 and enrolled in the Inova Translational Medicine Institute's clinical study entitled “Molecular Study of Pre-term Birth.” All study participants provided written informed consent for use of their genome sequences for research purposes. The “Molecular Study of Pre-term Birth” was approved by the Institutional Review Board of Inova Health System and the Western Institutional Review Board (#1124761). The analyses reported here were part of an investigation of the role of cancer-susceptibility genes in the etiology of pre-term birth, an area of research motivated by the similarities between pregnancy and malignancy [6], [7].

### Participants

The cohort for this analysis consists of 681 adults from 352 families, comprising 337 men aged 18–50 (median 34) and 344 women aged 18–44 (median 32). None of the individuals are first degree relatives, as confirmed by genomic analysis. The country of birth of the subjects and their parents were self-reported. The cohort is representative of the population of Northern Virginia and of the population giving birth at Inova Fairfax Hospital by race, ethnicity, and socioeconomic status [8]. Approximately one third of the subjects (34% of the men and 35% of the women) were enrolled in the study as parents of a pre-term neonate, and two thirds as full term controls. No significant association between the cancer gene variants and term status was found.

Self-report questionnaires and hospital medical records were reviewed for cancer status. Three individuals had a cancer diagnosis prior to enrollment: one man with renal cancer, one man with cancer of an unknown type, and one woman with breast cancer. None of the participants reported a personal and family history indicative of a highly penetrant cancer-predisposing germline mutation, namely early age of onset and/or multiple affected family members.

### Samples and sequencing

Whole blood samples were collected from all subjects in BD Vacutainer K2-EDTA tubes. Genomic DNA extraction was performed on the QiaSymphony automated DNA extractor using the DNA Midi kit (QIAGEN Inc., Valencia, CA). Samples were sent to Complete Genomics (Mountain View, CA) for whole genome sequencing, assembly, and variant calling [9], [10]. Sequencing was performed with the DNA nanoball array technology. Genome sequences were assembled with Complete Genomics' Assembly Pipeline versions 2.0.0-2.0.3 using the NCBI build 37 (hg19) human genome reference assembly [11]. Coverage statistics were calculated using weight-sum sequence coverage depth. On average, 70% of each genome and 80% of each exome had >40x coverage. Variants from the masterVar files from all genomes were merged into a single VCF v4.1 file with mkvcf (beta) from the CGA tools suite, version 1.6.0.

Gene annotations were computed with a modified version of the GLU software package, version 1.0b3-prerelease4 [12], using genome coordinates of exons, transcripts, and coding regions from the UCSC Genome Browser knownGene table [13]. Predicted protein sequence changes were calculated by translating the coding region of each transcript and the reference. Additional annotations from dbSNP 137 [14], COSMIC version 65 [15], HGMD Professional 2012.3 (BIOBASE), and PolyPhen-2 [16], [17] were added using the ANNOVAR tool [18]. PolyPhen-2 scores >0.85, between 0.85 and 0.15, and <0.15 were coded as “probably damaging”, “possibly damaging”, and “benign”, respectively [17].

### Quality filtering

Genotype calls were filtered for reliability using a predictive model trained on 341 randomly selected cancer-gene variants which were validated by Ion Torrent sequencing. Model building was performed with weka-3–6 [19] using default parameters except as noted. Attributes were selected by the BestFirst algorithm from the genotype quality information provided by Complete Genomics. Filtering parameters were determined using the J48 decision tree algorithm with 10-fold cross-validation. The resulting model incorporates two types of filters: a position filter and a genotype filter. The position filter excludes all variants at genomic locations with an overall call rate across the cohort of <80% or with an average fractional allele depth ≤0.295. The genotype filter masks calls with a minimum allele depth ≤11. Based on 10-fold cross-validation, the error rates for genotypes passing these filters were estimated to be <1.3% for false negatives and <2.3% for false positives.

### Genes and variants

The Cancer Gene Census, a curated collection of 487 genes with mutations causally implicated in oncogenesis from primary patient samples [20], was downloaded from the Sanger Center website (9/2012). To focus on variants that could impact cancer susceptibility due to predicted protein-sequence changes, we excluded genes for which the causal link to cancer was aberrant expression rather than mutation, keeping only genes listed in the Census due to missense, frameshift, splicing, or nonsense mutations. We included both genes with known cancer-predisposing germline mutations, as well as genes for which only somatic oncogenic mutations are currently known, since germline variation in genes with somatic mutations can also affect cancer susceptibility [20]. Loci omitted from or ambiguously mapped to the reference assembly were excluded, leaving 158 genes of interest.

Variants are defined as sequence differences from the reference, as calculated by the WGS pipeline. A variant was categorized as frameshift, nonsense, or splice-site disrupting if it had that predicted effect on any of the annotated transcripts associated with a cancer gene. Allele frequencies were computed from the called genotypes. Rare variants are defined as variants with minor allele frequency (MAF) <1%, and common variants those with MAF >5%.

The coding length of a gene is defined as the total number of bases predicted to be translated in any of the associated transcripts. Rates of per-gene variability, represented as the number of variants per kilobase (kb), were computed as the slope of the regression line of the number of variants in each gene on coding length.

Results from the per-gene analyses are presented for a set of five key genes as examples of the findings from all 158 genes. These genes were selected since they are well-known cancer genes that can carry clinically relevant mutations. The 5-gene set includes both small proteins with few variants and large proteins with many variants, and both tumor suppressor genes and oncogenes.

### Assignment of pathogenicity and return of results

Variants were classified as pathogenic if there were: (1) multiple primary reports of pathogenicity, (2) no reports with evidence against pathogenicity, and (3) molecular data demonstrating a detrimental effect. Pathogenic variants from study participants who consented to return of results were validated by Sanger sequencing and then reported to the multidisciplinary incidental findings committee for evaluation and communication to the individual's physician of record.

### Ancestry labeling and allele frequency analyses

Admixture coefficients were estimated for each subject with ADMIXTURE [21] using the procedure described by Libiger and Schork [22]. Allele frequencies for 6 ancestral populations - African, European, Native American, East Asian, Central Asian, and Oceanic - were computed with a reference panel comprised of 16,443 single-nucleotide polymorphisms (SNPs) [22]. To assign the individuals in our cohort to subpopulations, subjects were clustered based on their calculated admixture coefficients. The ancestry represented by each cluster was defined as the geographic region of the self-reported country of birth of the majority of individuals, excluding the United States. The African and African-European clusters are distinguished by the degree of admixture, with the African cluster closer to the African ancestral population. Ancestry groups were defined only for clusters with at least 20 individuals in order to calculate allele frequencies in increments of 5% or less for all genomic positions including those on the sex chromosomes. Smaller clusters were aggregated into an “Other” group, which was excluded from allele frequency calculations since it does not represent an ancestry-based population. For the other 6 subpopulations, statistically significant differences in MAF were computed by either the chi-squared test or Fisher's exact test. The chi-squared test was used for variants for which all expected values were >1, and the Fisher's exact test with simulated p-values was used for all other variants [23]. Variants for which the major allele in one population is the minor allele in another population are those for which the minimum frequency in any group is <0.5, the maximum frequency is >0.5, and both values are significantly different from each other and from 0.5 by one-sided Fisher's exact tests. For all statistical tests, p-values <0.05 were deemed significant.

### Additional software and databases

Statistical analyses were performed with R version 2.15.0 [24]. VCFtools 0.1.10 [25] and PLINK version 1.07 [26] were used to pre-process the variant data for the admixture calculation. Protein structures were displayed with Jmol [27]. The ClinVar database version 2013-8 [28], an archive of relationships between variations found in patient samples and phenotypes, was consulted for reports of clinical significance. In addition, the Breast Cancer Information Core (BIC) (version: 2/20/13) was examined for clinical reports of the *BRCA1* and *BRCA2* variants.

### Data availability

All variants reported in this publication are listed in Table S1 in File S1 and have been deposited in ClinVar with accession numbers SCV000083899 - SCV000086586. Researchers interested in sharing the genomic data are invited to contact the corresponding author.

## Results

### Cancer-gene variants are prevalent in a general population

To study the genetic variation in cancer-susceptibility genes in a cohort representative of a general, ancestrally diverse population, we analyzed whole genome sequences from participants in a pre-term birth research study. The cohort is comprised of 681 generally healthy adults of reproductive age, 49% men and 51% women, none of whom reported a personal and family history indicative of highly penetrant cancer-predisposing germline mutations.

We used this cohort to profile the germline variation of a set of 158 genes for which protein-sequence changes are causally implicated in oncogenesis. The coding regions of these 158 genes are well-covered in the genomic data, with an average per-gene coverage of 58x (range: 21x-84x), and with 99.99% of the positions sequenced in >10 individuals (Figure S1). This level of coverage is sufficient for high-quality variant calling but not clinical diagnosis [29]. We focused on small, nonsynonymous variations – substitutions, insertions, and deletions – since germline variations in the cancer-susceptibility genes are mostly of this type [20].

Among the 681 subjects we observed 2,688 predicted protein-affecting variants in the 158 cancer-susceptibility genes (Table S1 in File S1). Most of the variants are very rare – 65% are found in only a single individual and 75% are in 2 or fewer, with MAF <0.22%. Recent studies on variation in whole exomes [30] and in gene families [31] also found a majority of rare variants. Rare variants are thought to contribute significantly to the etiology of common disease [32], and strategies for prioritizing disease variants from WGS often include a frequency filter to exclude common variants. Forty-three percent (43%), or 1,166, of the variants are novel (not in dbSNP), all with MAFs between 0.07% and 1.4%. These data support the assertion that nearly all of the common variants in populations related to those in the 1000 Genomes Project have been discovered but that many rare variants are yet to be identified [33].

### Healthy individuals carry multiple cancer-gene variants

**Figure 1:**
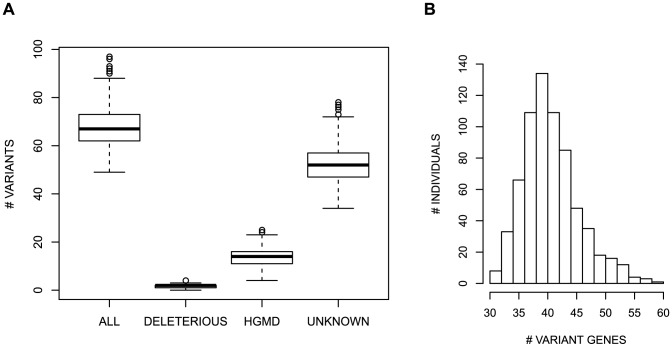
Profile of the variability per individual. (A) Boxplot of the total number of variants, the number of variants listed in HGMD, the number of likely deleterious variants, and the number of variants of unknown significance per individual for cancer-associated genes. (B) Distribution of the number of cancer genes with at least one nonsynonymous variant per individual.

Every individual in the cohort carries multiple nonsynonymous variants in the cancer susceptiblity genes, with an average of 68 variants per person (range: 49–97) (Figure 1A), and 99% of the individuals carry rare variants (median: 6 rare variants, range: 0–32). None of the participants have variants in all 158 genes; instead, the variants are distributed over a subset of 30–59 genes (median = 40) (Figure 1B) which varies by individual (see below). For an indication of whether these variants may be clinically relevant, all variants were assigned to three nonexclusive classes based on annotations related to potential impact on cancer susceptibility: (1) variants listed in HGMD as possibly disease-associated, (2) variants likely to have a deleterious effect on protein function, namely frameshift, nonsense, and splice-site variants, and (3) all other nonsynonymous variants. We use the latter class to represent variants of unknown significance (VUS), with the caveats that the clinical impact of some variants may be known but not captured in HGMD, and that variants assigned to the HGMD and deleterious classes may also have unknown effects on cancer susceptibility. Overall, 80 variants observed in the cohort were classified as deleterious (22 nonsense, 42 frameshift, 16 splice-site disrupting), 326 were annotated as possibly disease-associated in HGMD, and 2,297 are VUS (Table S1 in File S1). The study subjects have an average of 14 HGMD variants (range: 4–25), 2 variants in the deleterious class (range: 0–4), and 52 VUS (range: 34–78) (Figure 1A). The numbers of variants in the three individuals reporting a past cancer diagnosis were not outlier values for any of the variant classes. Although it is possible that the deleterious variants result from sequencing or annotation error, finding apparently detrimental variants in healthy individuals is not unexpected [34].

### Allele frequencies of cancer-gene variants are ancestry-dependent

Allele frequencies can differ between populations and these differences can have important medical implications [35]. In order to determine whether any of the protein-affecting cancer-gene variants in our cohort differ in frequency between ancestry groups, we assigned each individual to a subpopulation using the genomic data. A panel of 16,443 markers representing 6 ancestral groups associated with European, African, East Asian, Central Asian, Native American, and Oceanic populations [22] was used to calculate admixture proportions for each individual. Approximately half (49%) of the individuals were assigned nonzero coefficients for multiple populations, reflecting varying degrees of admixture or genetic ancestry incompletely captured by the model. Subpopulations were defined by clustering the subjects on the calculated admixture proportions. Clusters with fewer than 20 subjects were aggregated into an “Other” group and include Middle Easterners, admixed Eurasians, and others of unknown background.

**Figure 2:**
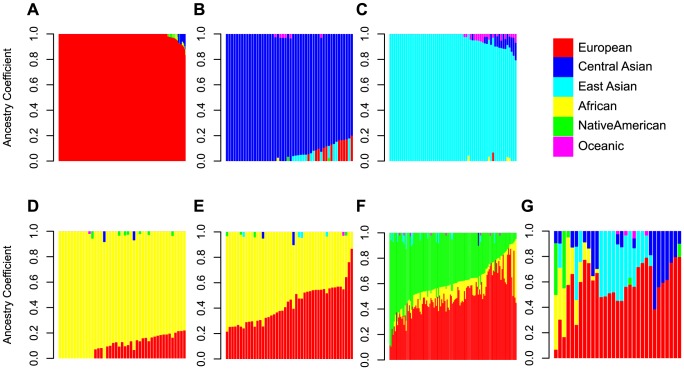
Admixture coefficients for the subpopulations. The admixture proportions of the 6 ancestral populations (colors) are displayed for all individuals in each of the 7 groups defined in the cohort (panels). (A) European (B) Central Asian (C) East Asian (D) African (E) African-European (F) Hispanic (G) Other. Red: European, Blue: Central Asian, Cyan: East Asian, Yellow: African, Green: Native American, Magenta: Oceania.

The seven resulting groups are listed in Table 1 and the admixture coefficients of the member individuals are plotted in Figure 2. For convenience, we use the names of the groups (European, African, etc.) to denote ancestral genetic background rather than geographic region of birth or ethnicity. The subpopulations correspond to 78–100% African ancestry for the African subpopulation, 79–100% East Asian ancestry for the East Asian subpopulation, 79–100% Central Asian ancestry for the Central Asian population, and 83–100% European ancestry for the European subpopulation. The cluster with individuals of 13–75% African ancestry and 21–87% European ancestry was named African-European. The admixture proportions distinguishing the African subpopulation (≥78% African) from the African-European group result from a breakpoint in the data and are comparable to the proportions of the dominant ancestry in the East Asian, Central Asian, and European groups (≥79%, ≥79%, ≥83%, respectively). The Hispanic subpopulation includes diverse admixtures of Native American and European ancestry with 0–50% African ancestry. These two- and three-way admixtures reflect the demographic history of Latin America [36].

**Table 1:** Ancestry-based subpopulations.

Subpopulation	# Individuals
African	43
African-European	46
Central Asian	50
East Asian	62
European	331
Hispanic	118
Other	31

**Figure 3:**
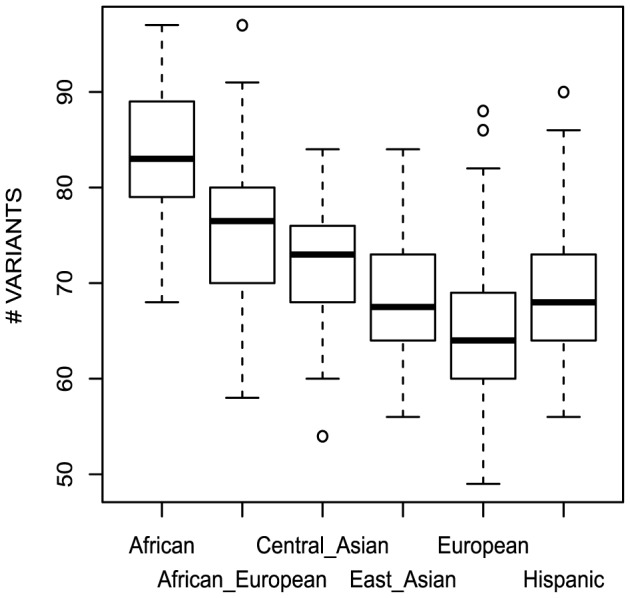
Number of cancer-gene variants per individual by ancestry. The distribution of the number of nonsynonymous genes per subject for each of the 6 ancestry-based subpopulations.

The ancestry-based subpopulations differ in the number of cancer-gene variants per person (Figure 3) (p<2.2e-16 by ANOVA). Europeans tend to have fewer variants (mean = 64.5) and Africans the most (mean = 84, 30% higher than Europeans), consistent with genome-wide estimates [37]. The number of variants in African-European individuals is intermediate between Africans and Europeans. The African, African-European, and East Asian subpopulations have about twice as many novel variants per person as Europeans, and Central Asians have threefold more (Table 2). The finding that Central Asians have more novel variants per person than Africans, who have higher total numbers of cancer-gene variants (Figure 3), may reflect a bias in the populations that have been sequenced and supports the efforts aimed at increasing the diversity of the populations sampled in sequence databases.

**Table 2:** Average numbers of cancer-gene variants per individual by ancestry.

	Total	Deleterious	HGMD	Novel
**African**	84.0	2.1	13.8	2.26
**African-European**	75.6	2.0	13.8	2.24
**Central Asian**	71.6	2.2	14.4	3.64
**East Asian**	68.6	1.8	12.0	2.73
**European**	64.5	1.8	13.9	1.19
**Hispanic**	68.3	1.9	13.2	1.70

The number of deleterious variants per individual is also significantly different between ancestral groups (p<4e-4 by ANOVA; Table 2). Averages range from 1.8 in Europeans and East Asians to 2.2 in Central Asians. For HGMD variants, there is also a statistically significant difference between the groups (p<9e-4 by ANOVA), with East Asians having the fewest variants on average recorded in that database (Table 2). However, the differences in the numbers of deleterious and HGMD variants are small and an association with ancestry needs to be examined in a larger cohort.

Differences between ancestry groups are also reflected in the allele frequencies of cancer-gene variants. Table S1 in File S1 lists allele frequencies in each of the population groups for the complete set of 2,688 variants. Fourteen alleles have frequencies >50% in all subpopulations (Table S2 in File S1), suggesting that the reference sequence carries a minor allele at those positions. We analyzed population differences in allele frequencies for common variants, since most rare variants are found in a single individual. Among the 223 variants with frequency >5% in any of the 6 ancestry-based groups, 216 have allele frequencies that differ between the subpopulations (Table S3 in File S1). Of these, 43% are found in all six subpopulations and 58 are specific to one of the four ancestry groups with lower degrees of admixture, 49 in Africans, 2 in Central Asians, 6 in East Asians, and 1 in Europeans. There are also 21 variants in which the minor allele in one population is the major allele in another (Table 3), of which ancestry-dependent frequencies have been recognized previously for at least 3, *ERBB2* c.3508C>G (p.Pro1170Ala) [38], *TP53* c.215C>G (p.Pro72Arg) [39], and *BRCA1* c.2612C>T (p.Pro871Leu) [40]. Little is known about the clinical significance of these 21 variants. Four, *TP53* c.215C>G (p.Pro72Arg) [41], *BRCA1* c.2612C>T (p.Pro871Leu) [42], *ERBB2* c.3508C>G (p.Pro1170Ala) [43], and *FLT3* c.680C>T (p.Thr227Met) [44], [45] have been linked to the development of cancer or to treatment response. However, these associations are typically of small effect or were derived from small samples; hence, more work is needed to establish a definitive relationship. If these associations are validated, they illustrate the importance of considering ancestry when selecting treatment options for patients.

**Table 3:** Positions at which the major allele differs between ancestry groups.

						Frequency of Nonreference Allele^a^							
Position	Ref	Alt	rsID	Gene	p	African	African-European	Central Asian	East Asian	European	Hispanic	Amino Acid Change	Protein ID
chr9:98209594	G	A	rs357564	PTCH1	4.41E-10	0.23	0.26	0.45	**0.64**	0.37	0.43	P1164L	Q13635
chr2:29416366	G	C	rs1881421	ALK	1.41E-22	**0.65**	**0.55**	0.48	**0.77**	0.36	**0.62**	D461E	A9YLN7
chr2:29416481	T	C	rs1881420	ALK	9.99E-30	0.29	0.29	0.44	**0.77**	0.25	0.46	K423R	A9YLN7
chrX:76937963	G	C	rs3088074	ATRX	2.03E-20	**0.90**	**0.64**	**0.51**	0.31	0.33	0.35	Q929E	P46100
chr10:88635779	C	A	rs11528010	BMPR1A	9.21E-40	**0.77**	**0.63**	0.24	**0.69**	0.24	0.39	P2T	P36894
chr17:41244936	G	A	rs799917	BRCA1	3.64E-25	**0.88**	**0.62**	0.49	0.43	0.32	0.34	P871L	P38398
chr15:40477831	G	A	rs1801376	BUB1B	1.65E-18	**0.85**	**0.67**	**0.52**	0.33	**0.71**	**0.72**	R212Q	O60566
chr17:37884037	C	G	rs61552325	ERBB2	6.41E-27	0.13	**0.53**	**0.65**	0.41	**0.68**	0.43	P1170A	P04626
chr16:89806347	A	T	rs7195906	FANCA	6.42E-40	**0.73**	**0.65**	**0.66**	**0.98**	0.38	**0.64**	I416N	B7Z6Y4
chr16:89836323	C	T	rs7195066	FANCA	5.22E-48	**0.63**	**0.59**	**0.65**	**0.98**	0.32	**0.62**	G809D	B4DRI7
chr16:89849480	C	T	rs2239359	FANCA	3.92E-23	**0.72**	**0.63**	**0.51**	**0.80**	0.38	**0.59**	G501S	B4DRI7
chr16:89866043	T	C	rs7190823	FANCA	1.40E-36	**0.66**	**0.58**	**0.65**	**0.98**	0.39	**0.65**	T249A	O15360-2
chr13:28624294	G	A	rs1933437	FLT3	7.17E-11	0.30	0.41	**0.68**	**0.73**	**0.61**	**0.54**	T227M	P36888-2
chr2:47739551	A	G	rs2303424	MSH2	1.55E-15	**0.62**	**0.60**	0.43	**0.73**	0.36	0.45	Q915R	E9PHA6
chr9:36840623	G	A	rs3780135	PAX5	2.03E-70	0.34	0.49	**0.85**	**0.94**	**0.95**	**0.88**	T293I	E7EQT0
chr8:145737816	C	T	rs4251691	RECQL4	2.27E-13	0.13	0.23	**0.63**	0.48	0.44	0.49	R1005Q	O94761
chr3:47125385	G	A	rs4082155	SETD2	7.03E-09	0.23	0.41	**0.63**	**0.59**	**0.53**	0.42	P2029L	Q9BYW2
chr1:2488153	A	G	rs4870	TNFRSF14	2.53E-12	**0.79**	**0.71**	**0.72**	0.44	0.47	**0.52**	K17R	Q92956
chr17:7579472	G	C	rs1042522	TP53	2.49E-18	0.37	**0.52**	0.41	**0.56**	**0.74**	**0.69**	P72R	P04637
chr14:81575005	C	A	rs3783941	TSHR	7.25E-07	**0.62**	**0.64**	**0.73**	0.39	**0.65**	**0.59**	R269S	NP_001136098
chr8:30999280	G	T	rs1801195	WRN	0.001403	0.43	**0.55**	0.42	**0.60**	0.46	**0.58**	L541F	Q14191

a Bold: Allele frequencies >0.5.

### Per-gene variation

**Figure 4:**
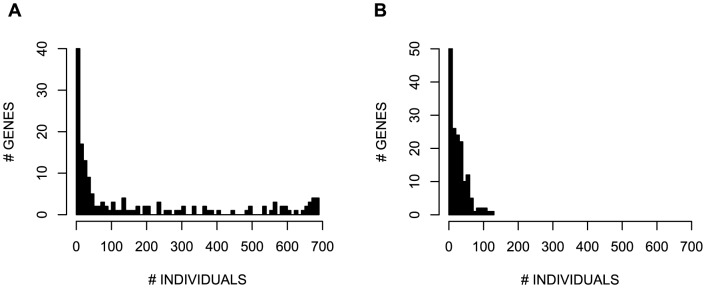
Variation prevalence per gene. Distribution of the number of individuals with a variant per gene for (A) all variants (B) rare variants.

Next we analyzed the variants on a per-gene basis to determine which genes are more or less likely to have variants reported from WGS of healthy individuals. The variant load for each of the 158 genes is listed in Table S4 in File S1. Four genes - *SRSF2*, *U2AF1*, *MAP2K4*, and *GNAQ* - have no nonsynonymous variants in our cohort, 36 genes have variants in fewer than 10 individuals, and 35 have variants in over half of the individuals (Figure 4A). Limiting the analysis to only rare variants, 154 (97.5%) of the genes exhibit variation in at least one individual (Figure 4B). On average, a cancer gene has rare variants in 4% of our population, with a range of 0% to 18% (0–125 individuals). Among the genes with rare variants in the most individuals are *BRCA1*, *BRCA2*, *APC*, *MLL2*, and *MLL3*, genes which are commonly mutated in cancers. *BRCA1*, *BRCA2*, and *APC* are well-studied because of the presence of frequent, pathogenic mutations. *MLL2* and *MLL3* have recently been discovered to be mutated in a wide range of tumor types [46], and the prevalence of the observed variation suggests they may warrant more in-depth study.

**Figure 5:**
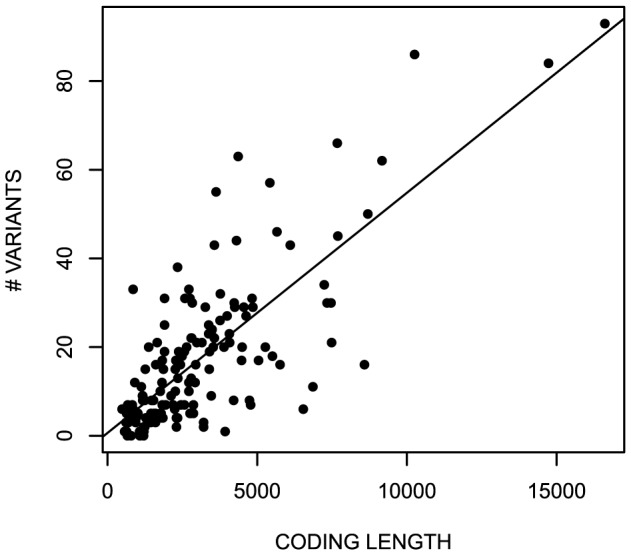
Correlation between the number of variants and coding length. The number of nonsynonymous variants vs. total number of coding bases for each of the 158 cancer-susceptibility genes.

The prevalence of the variation in each gene correlates with the number of variants. Sixty percent (60%) of the variability is accounted for by coding length (Figure 5), a trend previously noted for all single nucleotide variants exome-wide [30]. The overall rate of ∼6 variant positions per kb of coding sequence is comparable to the predicted variant discovery rate for a population the size of our cohort [31]. The most variable gene is *TNFRSF14*, with 39 variant positions per kb (Table S4 in File S1).

The per-gene variability may also depend on the type of cancer gene. Three types have been described: oncogenes, tumor suppressor genes, and predisposition genes [46]. The latter are genes for which germline mutations may predispose to cancer but which have few somatic mutations. Tumor suppressor genes and oncogenes have ∼5 variants per kb of coding sequence, whereas predisposition genes have ∼8 variants/kb, similar to the rate for all genes. The difference is statistically significant, with p<0.012 by ANCOVA. The lower rate of variability for oncogenes and tumor suppressor genes may indicate greater evolutionary constraint.

### Characterization of variation within key genes

In addition to population allele frequencies and literature reports of disease association, analysis of the effect each variant might have on the structure and function of the encoded protein can provide information pertinent to cancer risk prediction. We illustrate the gene-specific findings with a set of 5 well-known cancer genes of clinical relevance, *BRCA1*, *BRCA2*, *TP53*, *KRAS*, and *PTEN*. The variants and allele frequencies are listed in Table S1 in File S1.

### BRCA1 and BRCA2

*BRCA1* and *BRCA2* are the two major breast cancer susceptibility genes. Germline mutations in either of these tumor suppressor genes are associated with hereditary breast and ovarian cancer syndrome, which accounts for an estimated 2–8% of breast cancer cases worldwide [47]. In our cohort, 92% of the subjects carry nonreference alleles in one or both of these genes (excluding homozygous variant genotypes at chromosome 13 position 32929387 in *BRCA2* at which the reference sequence has a rare minor allele): 498 individuals with variants in *BRCA1* and 482 with *BRCA2* variants. Rare variants are also prevalent, with 27% of the population carrying rare variants in at least one of these two genes.

Most of the variants in these two genes are rare, with 83% of the 46 variants in *BRCA1* and 91% of the 86 variants in *BRCA2* having MAF <1%. The 4 common variants in *BRCA1*—c.2612C>T (p.Pro871Leu), c.3113A>G (p.Glu1038Gly), c.3548A>G (p.Lys1183Arg), and c.4837A>G (p.Ser1613Gly)—all show ancestry-dependent allele frequencies. Consistent with published data, the 871Leu-form is the predominant isoform in Africans whereas the Pro-form is more frequent in Europeans. Central Asians have the highest frequency of the other 3 common *BRCA1* variants. The 3 common *BRCA2* variants c.865A>C (p.Asn289His), c.1114A>C (p.Asn372His), and c.2971A>G (p.Asn991Asp), also exhibit significant differences between groups, with the highest frequency observed in Central Asians.

Almost all of the *BRCA1* and *BRCA2* variants observed in our cohort are unlikely to have strong effects on cancer susceptibility. For *BRCA1*, none of the variants were classified as pathogenic. One interesting variant is rs80356920, which introduces an isoform-specific stop codon into transcript 5 (NM_007299.3) and a missense change (valine to alanine) into transcripts 1–4 that is reported to reduce the protein's transcriptional activation function in vitro [48]. This variant, found in a single individual in our cohort, is of unknown clinical import according to BIC, and the possible deleterious effects at the molecular level make it a good candidate for future studies.

Missense mutations in *BRCA1* and *BRCA2* are the most difficult to classify clinically [49]. In our cohort, the missense variants in *BRCA1* are spread throughout much of the protein, whereas the cancer-associated mutations tend to cluster in the N-terminal RING domain and the C-terminal BRCT domain [50]. BIC reports 4 pathogenic mutations in the BRCT domain, of which 1 is in the phosphopeptide binding site and 2 are in the hydrophobic interface between the BRCT repeats [51]. In our cohort, we observed 4 missense mutations in the BRCT domain, c.4956G>A (p.Met1652Ile), c.5306A>G (p.Tyr1769Cys), c.5411T>A (p.Val1804Asp), and c.5504G>A (p.Arg1835Gln), all apparently in less critical locations of the encoded protein. Three of these are predicted to have benign effects by PolyPhen, and one, p.Arg1835Gln, is predicted to be probably damaging. No mutations in the central RING domain structure were observed in the cohort.

For *BRCA2*, 2 variants observed in our cohort are splice-site or nonsense variants. The nonsense mutation rs11571833 (p.Lys3326Ter) introduces a stop codon toward the 3′ end of the transcript. Although truncating mutations are often deleterious, this variant is not likely to be strongly pathogenic since the mutation is near the C-terminal end of the protein, consistent with the 301 reports in BIC listing this variant as not clinically important. Results from a recent meta-analysis of GWAS studies suggest that this variant is associated either with slightly higher risk of breast cancer or is in linkage disequilibrium with higher risk variant(s) [52]. In contrast, c.8487+1G>A (rs81002798) was classified as pathogenic. This variant, found in a single individual in our cohort, is a splice-site-disrupting mutation shown to affect RNA splicing in vitro [53] and has 7 reports of pathogenicity in BIC.

The *BRCA2* missense variants in the cohort are located throughout the protein, but are notably absent from the 8 RAD51-binding BRC repeats except for one instance of a conservative change in repeat 8, c.6215C>G (p.Ser2072Cys), predicted to be possibly damaging by PolyPhen. There are 19 missense variants within the C-terminal DNA-binding domain. Based on the crystal structure of murine BRCA2 [54], the variants occur in all five domains that comprise the DNA-binding domain, and six variants are in the disordered N- and C-terminal regions. Only one variant is at a position that aligns with a murine residue implicated in binding one of this domain's ligands, single-stranded DNA, double-stranded DNA, or the DSS1 protein. This variant, c.8187G>T (p.Lys2729Asn), is predicted to be probably damaging by PolyPhen and has somewhat reduced homology-directed repair activity in vitro, but the degree of activity is significantly greater than that of BRCA2 constructs with known pathogenic mutations [55].

### TP53

*TP53* is the most frequently mutated cancer gene in tumor samples. Somatic mutations in this tumor suppressor are found in a wide range of tumors, and germline mutations can cause Li-Fraumeni syndrome, a condition leading to a high, early-onset risk of multiple types of cancer. In our cohort, we observed 15 missense variants and no nonsense or frameshift variants. Among the missense variants are the two most-studied polymorphisms, c.215C>G (p.Pro72Arg) and c.139C>T (p.Pro47Ser). Allele frequencies for both these variants are ancestry-dependent. In our cohort, the frequency of the arginine-encoding form of codon 72 is 74.1% in Europeans and 37.2% in the African subpopulation, in agreement with published frequencies of up to 72-83% and 37%, respectively [39]. The second polymorphism, c.139C>T (p.Pro47Ser), is a rare, African-specific variant [56] that is present in 2 individuals in our cohort, both with African ancestry. Both the c.215C>G (p.Pro72Arg) and c.139C>T (p.Pro47Ser) SNPs have functional effects at the molecular level, but their clinical association with cancer susceptibility is unclear [57].

**Figure 6:**
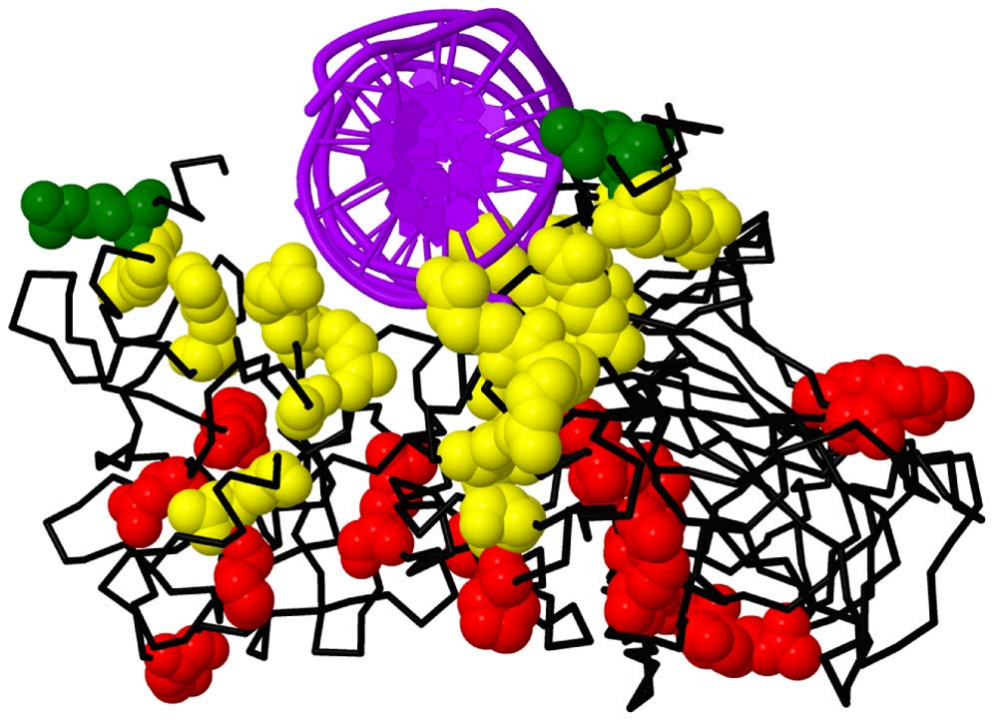
p53 DNA-binding domain variants. The DNA-binding domain of the p53 protein (black) bound to DNA (purple) [84]. Common somatic mutations (yellow) contact the DNA or stabilize the structure. Variants in our cohort (red) occur at residues distal to the DNA binding site except for Arg 283 (green).

Six of the other *TP53* variants observed in our cohort lie in the DNA-binding domain, the protein domain containing the majority of cancer-associated missense mutations. The most prevalent tumorigenic mutations occur at residues that directly contact DNA or stabilize the structure. One variant in our cohort, c.847C>T (p.Arg283Cys), occurs at a DNA-contacting residue and was previously reported as cancer-associated [58]. Although not satisfying the criteria for pathogenicity, this variant is an excellent candidate for further studies as to its clinical impact. The other 5 variants, at codons 110, 191, 202, 213, and 235, fall on the portion of the protein distal to the DNA-binding site, and are likely to have weaker effects on protein function and hence cancer risk (Figure 6). PolyPhen predicts c.329G>A (p.Arg110His) and c.605G>A (p.Arg202His) to be benign, and c.572C>G (p.Pro191Arg), c.638G>A (p.Arg213Gln), and c.704A>G (p.Asn235Ser) to be probably damaging.

The remaining 7 variants, all rare, lie in the transactivation and C-terminal regulatory domains or in putative alternative exons and are of unknown significance. Variants in the regulatory positions, such as phosphorylation sites and protein interaction sites in the N- and C-terminal regions [56], are lacking and only a single variant is seen in dbSNP. This suggests that in addition to mutations at these sites being insufficient for carcinogenesis, residues at these positions may be critical for normal function of the protein.

### KRAS

*KRAS* is an oncogene activated in a wide array of tumors, and mutations in this gene are predictive of response to epidermal growth factor receptor drugs. Mutation of a single residue, most often Gly12, is sufficient for oncogenic activation. In our cohort, we observed 3 *KRAS* variants, c.565A>C (p.Met189Leu) in two individuals, and c.535G>A (p.Gly179Ser) and c.531_533del in one individual each. All 3 variants lie in the coding region of one of two recognized isoforms of *KRAS*, c.565A>C (p.Met189Leu) and c.535G>A (p.Gly179Ser) in isoform a, and c.531_533del in isoform b. The three variants are in the C-terminal hypervariable regions of their respective proteins, which include two motifs required for membrane localization: a CAAX farnesylation motif and a palmitoylation site in isoform a, and a CAAX farnesylation motif and a lysine-rich polybasic domain in isoform b [59]. Met189 is at the variable “X” position of the farnesylation motif and is proteolytically removed during protein maturation. p.Gly179Ser is a residue of unknown function adjacent to the palmitoylated cysteine. The c.531_533del variant deletes one lysine from the hexalysine stretch of the polybasic domain, which remains capable of plasma membrane binding with mutation of up to 4 lysines [59]. Despite their location in a region of critical function, the variants are at positions apparently tolerant of variation and are unlikely to be pathogenic. Both SNPs are predicted to be benign by PolyPhen, and all 3 variants are annotated as either probably not pathogenic (c.535G>A; p.Gly179Ser) or of unknown clinical import in ClinVar. However, their effect on cancer susceptibility is unknown.

### PTEN

The tumor suppressor *PTEN* is one of the most frequently mutated genes in human malignances, and germline mutations are associated with the *PTEN* hamartoma tumor syndrome (which includes conditions such as Bannayan-Riley-Ruvalcaba syndrome and Cowden syndrome). In our cohort we observed a single variant, c.882T>G (p.Ser294Arg), in one individual. Residue 294 is located in an unstructured loop within the C-terminal domain of the protein [60]. This site has no mutations reported in COSMIC, HGMD, or ClinVar, is predicted to be benign by PolyPhen, and is of unknown function. The rarity of germline variants in this gene in a healthy cohort contrasts with the high frequency of somatic mutations in cancer patients and is consistent with both the small size of the encoded protein and its essential functions in critical signaling pathways [61].

## Discussion

Whole genome sequencing is becoming increasingly available in clinical practice, particularly with application to the diagnosis and prognosis of cancer patients. However, the use of WGS for assessment of cancer risk in the general population could strongly benefit from a better understanding of the clinical significance of many of the genetic variants in cancer genes. We began to address this need by characterizing the genetic variation in cancer-susceptibility genes present in healthy individuals, information that is critical for interpreting cancer-susceptibility risk from personal genome sequences [62].

We found that variation in cancer genes is prevalent. Based on our cohort, WGS of a healthy individual has a 100% chance of documenting multiple, protein-affecting variants in cancer genes. The extent to which the observed alleles contribute to cancer susceptibility is almost completely unknown. Since the study participants do not have clinical features consistent with carrying high-risk cancer-predisposing germline variants, most of the alleles identified are unlikely to represent highly penetrant, pathogenic mutations. Yet given the prevalence of cancer in the United States [63], about 40% (272) of these 681 individuals will eventually develop cancer. This is consistent with the polygenic model which proposes that an individual's cancer risk is the combined effect of multiple variants, each with relatively small effect on risk or protection and with different degrees of penetrance, and environmental factors [64].

Cancer risk prediction methods model the likelihood of an individual developing cancer based on demographic factors, family history, environmental risk factors, and/or biomedical test data [65], [66]. With the availability of genotype data, cancer risk prediction methods incorporating genetic marker information are being developed, for which ancestry-aware allele frequencies can be an essential component [67]-[69]. Modeling the variants in the context of protein structure and function can also contribute to risk prediction since the variants in our generally healthy cohort tend to occur at positions tolerant of substitution. Such data are already being used to evaluate in silico function predictions of VUS [70]. As our knowledge improves, these data can also be applied to predicting disease development, progression, and treatment response, all of which can be influenced by germline variation [71].

In addition to providing a foundation for the interpretation of cancer risk, the allele frequencies can also contribute to the identification of disease-causing alleles by incorporation into meaningful prior probabilities of association between variants and disease [72]. Furthermore, variants in genes for which variation is infrequently observed may be flagged as an unusual finding that bears further scrutiny.

The allele frequencies are estimates that depend on the sampled individuals and the assigned ancestry labels and may differ from estimates derived from other individuals or ancestral groupings. A sampling of the allele frequencies in this cohort agrees well with genotyping results from the Human Genome Diversity Project [73], supporting the relevance of our estimates. Some of the variants with frequencies differing between subpopulations, such as *TP53* c.215C>G (p.Pro72Arg), were previously described as ancestry-dependent in gene-specific studies, further supporting the generalizability of the findings. The ancestry-based groupings are strengthened by using genomic data for clustering rather than relying on self-reported data. Methods under development for estimating local ancestry for each region of an admixed individual's genome will further improve allele frequency analyses [74].

One of the concerns about wide availability of WGS is the potential for incidental findings. How such findings should be handled by the medical community is currently under debate [75], [76]. Our results provide information pertinent to the discussion by demonstrating that sequencing the genome of a healthy individual has a >0.1% (1 in 681) chance of discovering a cancer-predisposing nonsynonymous mutation of known clinical importance. Although lower than the frequency of 5 of 573 individuals with high-penetrance cancer-susceptibility mutations observed in a recent study [3], the estimates are broadly consistent given the small number of pathogenic mutations and the differences in sample population composition. The non-negligible rate of discovery of variants with clinical consequences supports the need for the community to address the ethical, legal, and social implications of the technology.

A second concern about routine clinical application of WGS is the high likelihood of uninterpretable findings, since uncertain results can impact medical decision-making and increase costs [77]. Our data confirm that WGS of a healthy individual identifies multiple VUS in medically important genes. We observed an average of 68 nonsynonymous variants per individual, almost all of which are of unknown clinical significance. The number of VUS will decrease as more is learned about the relationship between sequence variation and phenotype, and as models for prediction of clinical impact improve. This is of particular import for rare variants, since they represent the majority of variants and are thought to contribute significantly to complex disease, yet their rarity makes association with phenotype difficult to determine [78]. Some of the genes most commonly mutated in cancer are also the most variable in this cohort, further confounding interpretation.

Interpretation of the results for cancer risk can strongly benefit from a more complete and accurate database of annotated variants. Problems with current databases include inconsistencies in annotation of disease-relevance between data sources, absence of known variants from public databases, and the predominance of variants without available clinical correlation. Several efforts are underway to address these needs, including ClinVar and the recently announced “global alliance,” created to facilitate sharing genetic and clinical data among medical researchers [79]. The 1,166 novel variants we identified here and made publicly available contribute to the efforts cataloging human genomic variation worldwide.

When WGS is performed, the variants are called by comparison to a reference sequence. However, if a variant is instead defined as the minor allele, use of the current reference sequence leads to both overcalling and undercalling of variants at positions at which the reference sequence carries a minor allele. The impact on variant calling also depends on the ancestry of the individual sequenced, since major alleles in the reference may be minor alleles in the population of interest. Table 3 and Table S2 in File S1 list the positions affected in the cancer-gene set, which can be used as a resource for adjusting the variant calls in a personal genome. Variant calling as well as genome assembly could be improved by the use of a reference sequence appropriate to the ancestry of the individual under study.

Our list of cancer-susceptibility gene variants is extensive but incomplete for four main reasons. First, the list of genes influencing cancer predisposition is limited by current knowledge and inclusion in source databases. Second, we intentionally examined only small, nonsynonymous variants but other variant types, including large deletions, amplifications, translocations, and synonymous changes, can also impact cancer susceptibility. Third, some variants are not captured for technical reasons, including limitations of the WGS technology [80] and issues with the reference genome assembly [81]. Fourth, although this cohort is large for a single WGS study, the number of individuals is too small to capture the full range of variation in these genes, particularly for underrepresented populations and rare variants. The variants themselves represent the genomes of adult individuals at the time of sample collection. We refer to these as germline variants; however, since somatic mutations occur over time [82] some of the variants may have been acquired rather than inherited.

Similar analyses to those reported here could have been performed with sequence data from other sources. We chose to study the pre-term birth cohort for three reasons: (1) the cohort includes individuals of European, African, Hispanic, East Asian, and Central Asian ancestry, (2) the genomes were sequenced at high quality (mean coverage 60x), and (3) the data were generated uniformly with a single technology and bioinformatics pipeline, a factor that is critical for avoiding the variability introduced when combining multiple datasets generated with different protocols [83]. Use of the pre-term cohort, with 34% of the genomes from parents of a pre-term baby, could introduce bias if variation in the cancer-predisposition genes influences prematurity. This possibility is under investigation but to date no relationship between pre-term birth and the cancer-gene variants has been elucidated. The consistency of our results with what is already known suggests that any such bias is limited.

The data reported here represent the first profile of germline variation in multiple cancer-susceptibility genes from WGS of a healthy, ancestrally diverse cohort. To our knowledge, this is the largest set of uniformly processed whole genome sequences from a single cohort. The results comprise a resource capturing cancer-gene variation in 6 ancestry-based populations, and define quantitative and qualitative expectations for the results of personal genome sequencing, whether whole genome, exome, or targeted sequencing.

## Supporting Information

Figure S1 **Sequence coverage.** Plot of the coverage for all coding bases in each gene for each individual. There are between 327,000 and 11,300,000 points plotted per gene, depending on total coding length. Genes are arranged by chromosome.
(DOCX)

File S1 **Supporting tables.** Table S1, Variants in cancer-susceptibility genes observed in the cohort. Table S2, Positions with a minor allele in the reference sequence. Table S3, Common variants with ancestry-dependent allele frequencies. Table S4, Per-gene statistics.
(XLSX)
