# Supplementary figures and images for: Germline Variation in Cancer-Susceptibility Genes in a Healthy, Ancestrally Diverse Cohort: Implications for Individual Genome Sequencing
Source: PLoS One. 2014 Apr 11;9(4):e94554. doi: 10.1371/journal.pone.0094554 (PMC3984285; doi:10.1371/journal.pone.0094554)

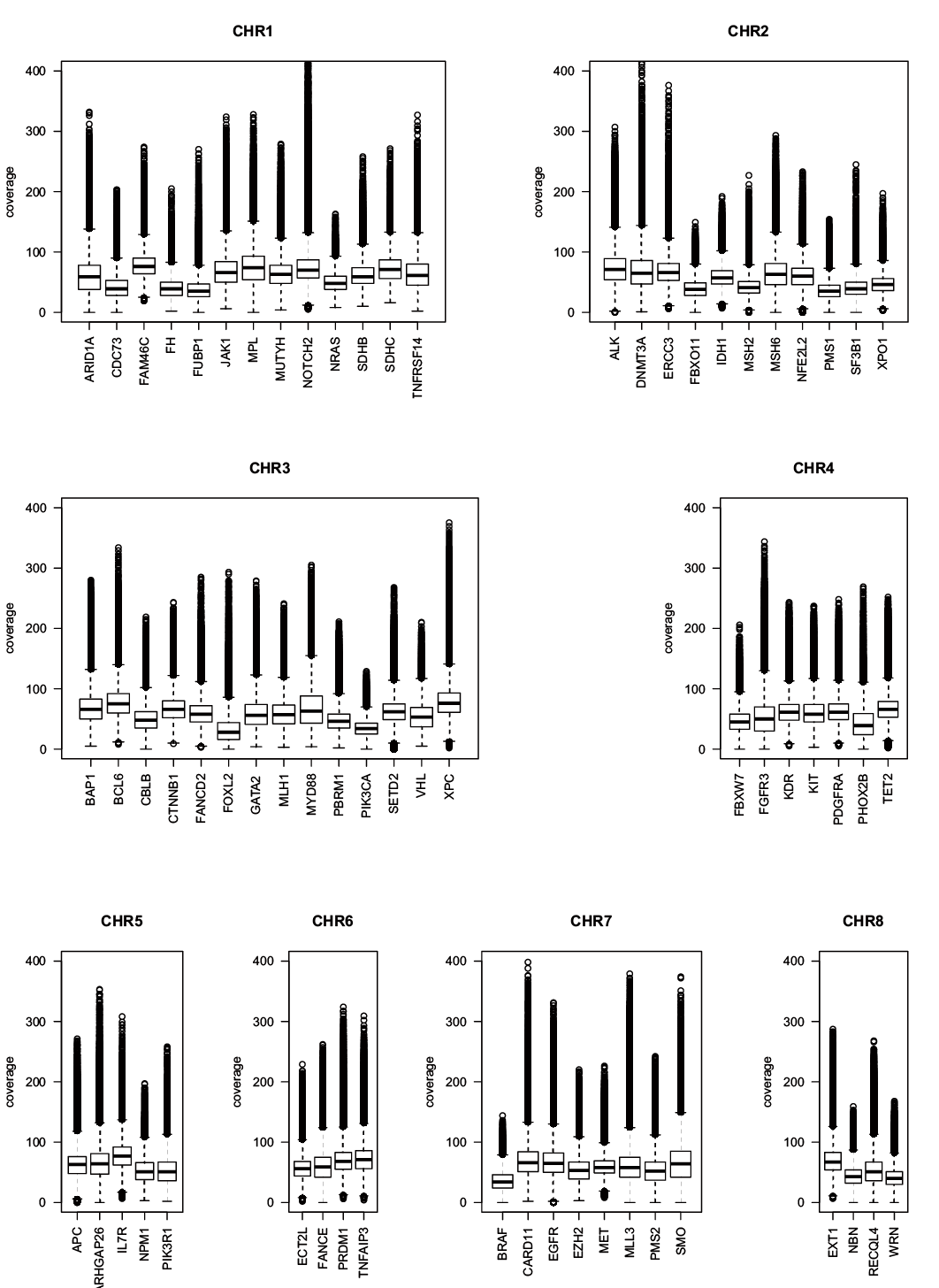


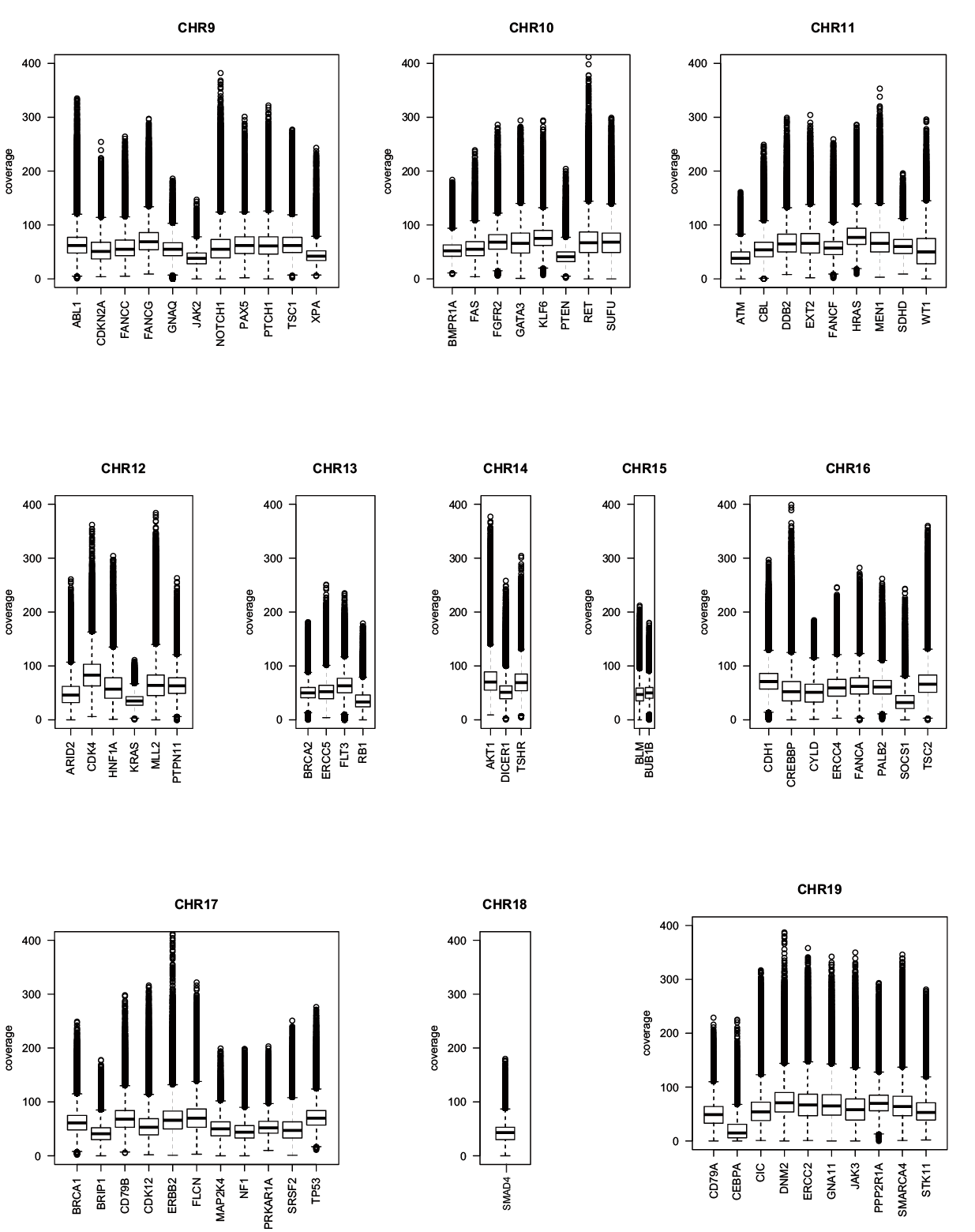


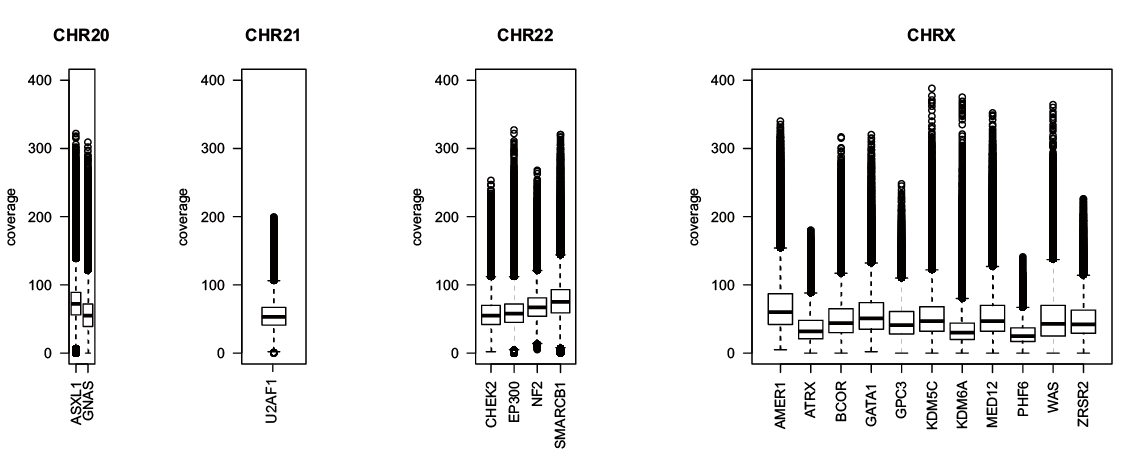

Supplement: Figure S1 — Sequence coverage. Plot of the coverage for all coding bases in each gene for each individual. There are between 327,000 and 11,300,000 points plotted per gene, depending on total coding length. Genes are arranged by chromosome. (DOCX) [file pone.0094554.s001.docx]
